# Adherence to End-of-Life Instructions Across Cognition Levels: Insights from the Health and Retirement Study

**DOI:** 10.1093/geroni/igaf122.2599

**Published:** 2025-12-31

**Authors:** Zahra Rahemi, Juanita-Dawne Bacsu, Swann Adams

**Affiliations:** Clemson University, Clemson, South Carolina, United States; Thompson Rivers University, Kamloops, British Columbia, Canada; University of South Carolina, Columbia, South Carolina, United States

## Abstract

Adherence to end-of-life instructions is understudied and may vary across cognition levels in older adults, as cognitive decline can impact decision-making and the implementation of advance care planning. This study aims to examine adherence to end-of-life instructions across different cognition levels in older adults. Data from deceased participants in the Health and Retirement Study (HRS, 1994–2018) were extracted using exit files to assess adherence to end-of-life instructions. Langa Weir classification was used for cognition categorization. Adherence was measured using three indicators: proxy-reported difficulties in following written EOL instructions, proxy-reported healthcare provider difficulties in following written instructions, and instances where the patient received unwanted treatment despite family or decision-maker refusal. Individuals with dementia and impaired cognition were older at death, less likely to be married, more likely to have a high school education or less, and predominantly female. They were less likely to discuss end-of-life care prior to death or have a living will at death (p < 0.05) and marginally less likely to want all possible care (p = 0.06). Regarding adherence indicators, dementia group participants were 2.37 times more likely to receive unwanted treatment despite family/decision-maker refusal compared to normal cognition participants (OR = 2.37 [95% CI: 1.04–5.40], p < 0.05). Our findings highlight disparities in adherence to end-of-life instructions, with individuals with dementia being more likely to receive unwanted treatment. These results underscore the need for improved communication between individuals involved in end-of-life care and strategies to ensure that end-of-life preferences are honored across cognition levels.

